# The Impact of Human Herpesviruses in Clinical Practice of Inflammatory Bowel Disease in the Era of COVID-19

**DOI:** 10.3390/microorganisms9091870

**Published:** 2021-09-03

**Authors:** Shuhei Hosomi, Yu Nishida, Yasuhiro Fujiwara

**Affiliations:** Department of Gastroenterology, Osaka City University Graduate School of Medicine, Osaka 545-8585, Japan; m2076030@med.osaka-cu.ac.jp (Y.N.); yasu@med.osaka-cu.ac.jp (Y.F.)

**Keywords:** human herpesviruses, herpes simplex virus, varicella-zoster virus, Epstein-Barr virus, cytomegalovirus, inflammatory bowel disease, ulcerative colitis, Crohn’s disease, SARS-CoV-2, COVID-19

## Abstract

Human herpesviruses (HHVs): herpes simplex virus (HSV) types 1 (HSV-1) and 2 (HSV-2), varicella-zoster virus (VZV), Epstein-Barr virus (EBV), cytomegalovirus (CMV), HHV-6, HHV-7, and HHV-8, are known to be part of a family of DNA viruses that cause several diseases in humans. In clinical practice of inflammatory bowel disease (IBD), the complication of CMV enterocolitis, which is caused by CMV reactivation under disruption of intestinal barrier function, inflammation, or strong immunosuppressive therapy, is well known to affect the prognosis of disease. However, the relationship between other HHVs and IBD remains unclear. In the transplantation field, reactivation of other viruses, such as HHV-6, could cause colitis under immunosuppressed condition. Recent research revealed that combined infection of some HHVs could be a risk factor for colectomy in patients with ulcerative colitis. This suggests that it would be important to clarify HHV behavior in the treatment for patients with IBD, especially in those under immunosuppressive therapies. Looking at the relationship with recently emerged novel coronaviruses (SARS-CoV-2), there are reports describe that SARS-CoV-2 might induce reactivation of HSV-1, EBV, VZV (herpes zoster), and HHV-6/7. If SARS-CoV-2 infection becomes common, vigilance against HHV reactivation may become more crucial. In this review, we discuss the impact of HHVs in clinical practice of inflammatory bowel diseases, especially during the SARS-CoV-2 pandemic.

## 1. Introduction

Crohn’s disease (CD) and ulcerative colitis (UC), which are chronic and relapsing inflammatory bowel disease (IBD), have been known to be complex multifactorial diseases, involving not only host factors, such as genetic factors, mucosal barrier dysfunction, and altered immune response, but also environmental factors, including infectious agents [1,2]. Recent papers have shown that alteration in the enteric virome could play a role in IBD pathogenesis [3,4]. The interactions between certain enteric viruses and Paneth cells could lead to the activation of the innate immune pathway and inflammatory immune response in IBD [5]. It has been demonstrated that enteric viruses could also infect macrophages stimulating the production of tumor necrosis factor alpha (TNF-α) and interferon (IFN)-γ [5].

The human herpesviruses (HHVs) are divided to three categories, based on biological characteristics: α-herpesviruses (which include herpes simplex virus (HSV) types 1 (HSV-1) and 2 (HSV-2), varicella-zoster virus (VZV)), β-herpesviruses (which include cytomegalovirus (CMV), human herpesvirus 6 (HHV-6), and human herpesvirus 7 (HHV-7)), and γ-herpesviruses (which include Epstein-Barr virus (EBV) and human herpesvirus 8 (HHV-8)) [6]. The complication of CMV enteritis in IBD, especially in UC practice, is believed to be caused by CMV reactivation as a result of disruption of intestinal barrier function, inflammation, or strong immunosuppressive therapy, and is known to affect the prognosis of UC [7]. However, the relationship between other HHVs and IBD remains unclear. In our previous study on HHV infection in the colonic mucosa of patients with IBD, we demonstrated that half of the CMV-infected patients had combined infections with EBV or HHV-6, and all the combined infections were cases of UC, indicating a higher rate of subsequent total colorectal resection in patients with mixed infections than in patients with only one type of HHV detected [8]. In the transplantation field, some reports have argued that HHV-6 and CMV infections interact [9,10,11], therefore, understanding the mixed infection status of HHVs may be important for the treatment of IBD. Looking at the relationship with new coronaviruses (SARS-CoV-2), there are reports suggesting that SARS-CoV-2 may induce reactivation of HSV-1, EBV, and HHV-6/7, and a case of herpes zoster (VZV reactivation) in a patient with coronavirus disease 2019 (COVID-19) [12,13]. If SARS-CoV-2 infection becomes common, vigilance against HHV reactivation may become more crucial. In addition, it is known that the risk of herpes zoster increases during administration of Janus kinase (JAK) inhibitors [14], which may require further attention and discussion of the need for a herpes zoster vaccine. In this review, we describe the impact of HHVs in patients with IBD, mainly in clinical practice.

## 2. α-Herpesviruses in Inflammatory Bowel Disease

### 2.1. Herpes Simplex Virus 1/2

The estimated seroprevalence of HSV-1 and -2 in the United States is approximately 50% and 15%, respectively [15]. About one third of the world population is thought to have experienced symptomatic HSV-1 at some point throughout their lifetime. HSV-1/2 is generally spread via direct contact with bodily secretions. After primary infection of epithelial cells, the viruses move to neurons of the peripheral nervous system as latent infection and can be periodically reactivated, resulting in recurrent clinical episodes. Although infection of the gastrointestinal tract is very rare, except for the esophagus [16], herpes simplex esophagitis is usually found in immunosuppressed patients, such as those with malignancies, those with acquired immunodeficiency syndrome (AIDS) [17], and those receiving immunosuppressive treatment [18,19]. In fact, HSV-1/2 were not detected in mucosal specimens and stool obtained from IBD patients by polymerase chain reaction (PCR) (Table 1) [8]. In the clinical practice for IBD, systemic steroids and antimetabolites could cause increased risk of HSV reactivation [20,21]. Although HSV colitis is known to be rare disease, several IBD patients who were treated with corticosteroids were reported [21]. A recent report has described cases of HSV hepatitis which developed in a patient with CD after treatment with anti-TNF-α therapy [22,23]. 

### 2.2. Varicella-Zoster Virus

The initial infection with VZV usually leads to acute varicella or chickenpox during childhood, then the virus persists in neurons of the dorsal root ganglia. Patients with VZV infection involving the gastrointestinal tract are very rare. Investigations of VZV infection of the intestinal mucosa of IBD showed that the virus was not detected by PCR [8], and reports on mucosal injury by VZV infection are limited [27,28]. However, clinicians should consider the possibility of herpes zoster cases, also called shingles, in clinical practice of IBD, because patients with IBD are known to be at increased risk (HR: 1.49, 95% CI: 1.42–1.57) for herpes zoster [29]. The immunosuppressed condition can lead to the reactivation of the latent VZV, resulting in herpes zoster. Postherpetic neuralgia occurs in 10–18% of herpes zoster cases, and this pain syndrome can last from months to years after the initial herpes zoster [30]. Ramsay Hunt syndrome, encephalitis, myelitis, and vasculopathy could also occur after herpes zoster as a complication [31]. A retrospective cohort and nested case–control study showed that the herpes zoster risk is further increased by use of corticosteroids (OR: 1.73, 95% CI: 1.51–1.99), thiopurines (OR: 1.85, 95% CI: 1.61–2.13), anti-TNF-α therapy (OR: 1.81, 95% CI: 1.48–2.21), and combination therapy of thiopurines and anti-TNF-α therapy (OR: 3.29, 95% CI: 2.33–4.65) [29]. Recently, JAK inhibitors, which block multiple cytokines via inhibition of the downstream of the JAK signal, have emerged for IBD therapy. Human antiviral defense relies on type I and II interferons, both of which signal via the JAK-1 receptor [32]. In fact, therapy with tofacitinib, one of the JAK inhibitors, has been identified independently to increase risk (incidence rates: 4.0, 95% CI 3.7–4.4) for herpes zoster, especially in Asian patients (incidence rate: 8.0, 95% CI 6.6–9.6 in Japan, 8.4, 95% CI 6.4–10.9 in Korea) [33]. Recent in vitro research has demonstrated mechanisms by which tofacitinib reduced the interferon-γ production, proliferation, activation, and C-X-C motif chemokine receptor 3 (CXCR3) expression of VZV-specific CD4^+^ T cells [34].

The risk of herpes zoster could be reduced by vaccination. A live-attenuated vaccine against herpes zoster (Zostavax ^®^) has been used to reduce the risk of getting herpes zoster [35] and zoster-related post herpetic neuralgia [36]. However, this live-attenuated vaccine is contraindicated in immunocompromised patients, such as patients with malignancies of bone marrow or the lymphatic system, primary or acquired immunodeficiency patients, and patients receiving immunosuppressive therapy [37]. Recently, a two-dose recombinant herpes zoster vaccine (Shingrix ^®^) containing a recombinant VZV glycoprotein E and AS01_B_ adjuvant system was developed for prevention of herpes zoster. Phase III clinical trials revealed high efficacy (more than 90%) in adults aged ≥50 years to reduce the risks of herpes zoster and postherpetic neuralgia [38,39]. Theoretically, this recombinant vaccine can be used for immunocompromised individuals. A recent prospective observational study [40] showed safety for IBD patients in which two thirds were immunosuppressed. This study also observed a low rate of IBD flare (1.5%) [40]. Even though these vaccines have such high efficacy, the vaccination rates for herpes zoster in a nationwide IBD cohort were still very low (20.96%) [41]. In the field of IBD, where use of several immunomodulatory therapies, including JAK inhibitors, is available, physicians should attempt to improve vaccination rates among the IBD population.

## 3. β-Herpesviruses in Inflammatory Bowel Diseases

### 3.1. Cytomegalovirus

Although the seroprevalence varies depending on the socioeconomic status of countries, 83% (95% uncertainty interval: 78–88) of the general population are seropositive for CMV [42]. The virus remains with the host latently in cells of the early myeloid lineage, including granulocyte–macrophage progenitors and CD14+ monocytes, and endothelial cells [43,44]. The latent CMV can be reactivated under immunocompromised status, such as patients with AIDS, organ transplantation, hematological malignancy, cancer therapy, and corticosteroid therapy [45], driven by inflammation [46]. TNF-α can directly stimulate firing of the CMV immediate–early promoter and CMV reactivation [44,47]. CMV colitis, which occurs most commonly in the gastrointestinal tract, can develop in patients with IBD, especially in UC, and this is associated with a significant clinical morbidity, such as a toxic megacolon, requirement of colectomy, and high mortality rate [48,49]. Regarding risk factors for CMV colitis in UC, these include: higher age, higher activity of mucosal inflammation, and use of immunosuppressive treatment, including corticosteroids and thiopurines, but not TNF-α inhibitors [7].

**Figure 1:**
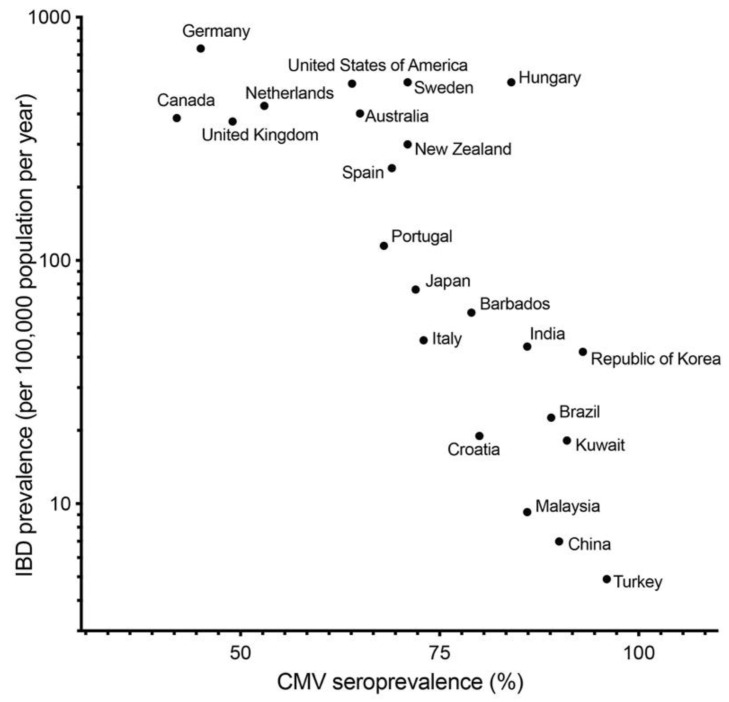
Relationship between seroprevalence of cytomegalovirus and prevalence of inflammatory bowel disease.

CMV infection can be associated not only with activity of UC and treatment, but also initiation and exacerbation of UC. In fact, several papers showed that colonic mucosa of UC patients have higher prevalence of CMV DNA than that of normal and CD patients [50,51,52]. In our previous study, we described that the prevalence of CMV DNA in colonic mucosa in patients with UC (13.9%) was higher than that in the control group (3.4%) and patients with CD (0%) [8]. As seroprevalence of CMV in developed countries is generally lower than in developing countries, the seroprevalence of CMV [42] and prevalence of IBD [53] are negatively correlated (Figure 1). This correlation indicates that CMV infection per se is not a key etiology of IBD.

CMV infection is clinically diagnosed based on positive serology, histopathological detection of owl’s eye inclusions and/or immunohistochemically stained cells, or PCR for CMV DNA in clinical samples such as blood, stool, or colonic mucosal specimens. PCR for CMV detection is reported to be more sensitive than immunohistochemistry [54,55]. Therefore, quantitative real-time PCR performed for samples of colonic mucosa would be a useful assay for accurate diagnosis of CMV colitis [56]. The therapeutic strategy for CMV infection in flared UC is still controversial. It is not easy to determine whether detected CMV in inflamed colonic mucosa is a pathogen or bystander. In fact, some studies showed the efficacy of antiviral treatment with ganciclovir or foscarnet for CMV infection in active UC patients, but others did not. A meta-analysis of 15 studies showed that antiviral treatment benefited only a subgroup of UC patients who were refractory to corticosteroids, whereas no benefit was observed in the overall UC population [57]. It is proposed that UC patients with high viral load detected by quantitative PCR in colonic mucosa should be a reasonable indication of antiviral treatment from recent studies; the majority of patients with a high viral load had a response to antiviral treatment [58] and high viral load in colonic mucosa predicted the failure of corticosteroids and short-term risk of colectomy [59].

The data of cytomegalovirus (CMV) seroprevalence and inflammatory bowel disease (IBD) prevalence were collected from publications by Zuhair M et al. [42] and Ng SC et al. [53], respectively; y = −10.47x + 994.4, *R*^2^ = 0.5154, *P* = 0.0002. 

### 3.2. Human Herpesvirus 6/7 

HHV-6 consists of two closely related variants, HHV-6A and HHV-6B. While HHV-6A has been poorly epidemiologically characterized, HHV-6B is known to infect more than 90% of the human population [60] and was reported to be the causative agent of roseola infantum, leading to febrile seizures in more than 10% of acute infections [61,62]. Both HHV-6A and HHV-6B establish a latent infection in their hosts [63,64].

Studies of HHV-6-associated colitis are very limited [65,66], although HHV-6/7 reactivation can cause encephalitis in immunocompromised patients [67]. Our previous study showed that HHV-6 was detected in 9.2% of colonic mucosa samples of IBD patients; however, there was no difference in the prevalence among UC patients, CD patients, and the healthy controls [8]. Moreover, no significant risk factors associated with HHV-6 infection were found in the logistic regression analysis, indicating that HHV-6 infection in the colon is not related to mucosal inflammation in IBD [8]. 

## 4. γ-Herpesviruses in Inflammatory Bowel Diseases

### 4.1. Epstein-Barr Virus

EBV infection is established in childhood or adolescence, and more than 90% of adults worldwide are seropositive for EBV [68]. EBV primarily enters into the host B cells and epithelial cells, after which the virus remains latent until reactivation. Patients under immunosuppressive therapies are at higher risk of lymphomas, especially posttransplant-like lymphomas, which might be related to a reactivation of a chronic EBV latent infection [69]. As patients receiving hematopoietic stem cell transplantation are also known to be at high risk of reactivation of EBV, close monitoring of EBV viral load has been recommended [70]. In patients with IBD, elderly patients and those taking infliximab are a risk factor for the presence of EBV DNA in blood [71,72]. On the other hand, several studies focusing on CD described that treatment with azathioprine and/or anti-TNF-α antibodies did not influence the EBV viral load [73,74]. This might be related to the prevalence of EBV in intestinal mucosa; 21.2% of patients with UC and 9.3% of patients with CD were positive for EBV DNA by multiplex PCR [8] (Table 1). This study also revealed that the existence of mucosal inflammation and use of corticosteroids, cyclosporine A, or tacrolimus were significant risk factors for EBV infection by univariate logistic regression analysis.

As gastrointestinal tract involvement of chronic active EBV mimics IBD, especially CD, it could be difficult to diagnose accurately [75,76]. This is another aspect of EBV to note in the gastrointestinal tract.

### 4.2. Human Herpesvirus 8

HHV-8 is also known as Kaposi sarcoma-associated herpesvirus (KSHV), and its seroprevalence is about 5–20% [77]. The most common manifestation of Kaposi sarcoma is skin lesions; however, in some cases, it can affect multiple organs, including gastrointestinal lesions. Although Kaposi sarcoma is commonly associated with immunosuppression from human immunodeficiency virus (HIV) or chronic use of transplant anti-rejection medications [78], iatrogenic Kaposi sarcoma can occur in patients using immunosuppressive therapies, mainly in kidney or liver transplantation recipient patients. There are several case reports describing HIV-negative Kaposi sarcoma in IBD patients treated with immunosuppressive therapies [79,80,81,82]. Although data on HHV-8 infection in IBD patients are limited, HHV-8 infection in gastrointestinal tissue is very rare; HHV-8 was not detected by mucosal PCR in 24 UC patients who underwent colectomy [83] and 41 IBD patients who underwent colonoscopy [26]. Therefore, Kaposi sarcoma in IBD might be very rare, however, knowledge of the existence of HHV-8-associated intestinal disease would contribute to accurate diagnosis.

## 5. Co-Reactivation of Human Herpesviruses

Some HHVs, such as CMV and EBV, are clinically relevant viruses and can be reactivated in immunodeficient, immunosuppressed, and immunosenescent states [84,85], therefore, co-reactivation of herpesviruses has been reported as clinically important. A recent study showed that older patients under high intensity immunosuppressive treatment and/or chemotherapy had a higher frequency of EBV co-reactivation with CMV viremia [86]. Co-reactivation of HSV and CMV was frequently seen among patients with severe acute respiratory distress syndrome (ARDS) [87]. Regarding patients with acute graft-versus-host disease (GVHD), co-reactivation of CMV and EBV was associated with poor prognosis after allogeneic stem cell transplantation [88]. In terms of IBD, we previously reported that combined infection of EBV or HHV-6 with CMV could be associated with pathogenesis of UC and poor clinical outcome [8]. We described that 5 of 11 (45%) and 1 of 11 (9%) UC patients with CMV also have co-existing EBV and HHV-6 infection, respectively, and a combined infection by EBV or HHV-6 with CMV is independently and significantly associated with an increased risk of colectomy in patients with UC [8]. In line with our study, Nahar S et al. evaluated the prevalence of HHVs in stool samples, which demonstrated a prevalence of CMV, EBV, and HHV-6 of 36.6%, 36.6%, and 11.3%, respectively, and a higher rate of co-existence of CMV and EBV and/or HHV-6 in patients with active UC (24.1%) [89]. HHV-6 could be a co-factor for the development of CMV infection in patients undergoing transplantation [10,11], and CMV primary infection could induce EBV reactivation [9]. These findings suggest that such interactions between CMV and other HHVs might promote mucosal inflammation in IBD, particularly in UC. Therefore, further basic and clinical research would be needed to clarify the role of co-reactivation of HHVs in IBD.

## 6. Human Herpesviruses in the Era of COVID-19

COVID-19, which is caused by SARS-CoV-2, can infect multiple organs, not only the lungs, but epithelial cells and the gastrointestinal tract [90]. Most individuals infected with SARS-CoV-2 have a mild to moderate disease course, while some of them progress to severe or critical disease, such as ARDS, pulmonary edema, cytokine storm, diffuse coagulopathy, and multiple organ failure [91], and require intensive care with mechanical ventilation/extracorporeal membrane oxygenation for respiratory support [92]. Therefore, therapies for moderate to severe COVID-19 require antiviral and immunomodulatory treatment, such as corticosteroids, anti-interleukin 6 receptor antibody, and inhibitors for the JAK pathway and spleen tyrosine kinase [93]. That distinct phenotype could be characterized by host immune responses. Hadjadj J et al. showed that impaired IFN type I activity, which was characterized by no IFN-β and low IFN-α production and activity, was associated with a persistent blood viral load, exacerbated inflammatory responses, and disease severity. These backgrounds can influence other viral infections. Several observational studies showed that patients with herpes zoster and pityriasis rosea, which is associated with reactivation of HHV-6/7, increased in number during the COVID-19 pandemic [12,13]. Critical COVID-19 patients were reported to develop EBV, CMV, and HHV-6 reactivations while in the intensive care unit (ICU) [94]. A suggestive clinical case report showed that a critical COVID-19 patient simultaneously worsened with reactivation of HSV-1 and VZV identified by next-generation sequencing from blood and sputum [95]. Co-reactivation of these herpesviruses, on the other hand, was reported to be associated with a prolonged mechanical ventilation duration, extracorporeal membrane oxygenation (ECMO) duration, ICU stay, and hospital stay [87]. These data collectively indicate that COVID-19 pneumonia could lead to a vicious cycle of co-reactivation of latent viruses and aggravation the COVID-19 disease course, and possibly severity of enterocolitis in IBD patients with COVID-19.

Another influence of the COVID-19 pandemic on other infections is a decline in regular check-ups and routine vaccinations. The childhood vaccination rate among 16-month-olds, which includes VZV, in Texas declined by 58% during the COVID-19 pandemic [96]. A cohort in Michigan also showed a reduction in childhood vaccination, especially in 5–16-month-olds, during the COVID-19 pandemic [97]. Efforts such as educating healthcare workers and parents are necessary to ensure that appropriate vaccinations continue to be provided during a pandemic.

The COVID-19 pandemic and lockdown, as several studies reported, could cause lifestyle changes and psychological stress [98,99,100,101]. In patients with IBD, lifestyle factors, such as smoking or drug adherence, and psychological stresses were reported to significantly influence the disease activity [102,103,104,105]. The pandemic and lockdown also resulted in reduced access to face-to-face outpatient clinics for IBD patients and diagnostic examinations, such as endoscopy [106,107]. Taken together, the pandemic and lockdown would change patients’ symptoms and the clinical course of IBD.

## 7. Conclusions

This review focuses on HHVs in IBD. It is well known that CMV can affect the clinical course of IBD, especially UC, however, the other HHVs are still not well understood. However, recent studies have revealed the possibility of novel influences from these viruses, such as interactions between CMV and other HHVs potentially promoting mucosal inflammation in UC, and this is an area that warrants further research. 

The worldwide pandemic and lockdown caused by COVID-19 has changed our lifestyle, resulting in changes in disease activity, clinical practice, and treatment for IBD patients. The COVID-19 pandemic could be also involved in HHV reactivation, which would influence the IBD clinical course. Therefore, if SARS-CoV-2 infections become more common, vigilance against HHVs in IBD care may become more important. At the same time, it may be necessary to discuss the need for a herpes zoster vaccine, as the COVID-19 pandemic has caused a decline in vaccination rates for various diseases.

## Figures and Tables

**Table 1:** Prevalence of human herpesvirus DNA in colonic mucosa of patients with inflammatory bowel disease.

Scheme	Location	Duration	Study Design	Cohort	Samples	Methods	Results
Van Kruiningen HJ et al., 2007 [24]	Belgium and France	Data unknown	Data unknown	CD: 70 Ctrl: 41	Surgical resection specimens	Conventional PCR	CD HSV-1: 0% HSV-2: 0% EBV: 15.7% CMV: 1.4% HHV-8: 0% Ctrl HSV-1: 0% HSV-2: 0% EBV: 7.3% CMV: 2.4% HHV-8: 0%
Ciccocioppo R et al., 2016 [25]	Italy	Data unknown	Prospective	Responder IBD: 30 Refractory IBD: 20 (UC: 35, CD: 15) Ctrl: 25	Colonic LPMCs	Real-time PCR	Responder IBD EBV: 66% CMV: 28% Refractory IBD EBV: 100% CMV: 48% Ctrl EBV: 28% CMV: 20%
Shimada T et al., 2017 [26]	Japan	2011–2015	Retrospective	IBD: 41 (UC: 33, CD: 8)	Mucosal biopsy samples from colon	Real-time PCR	IBD HSV-1: 0% HSV-2: 0% VZV: 0% EBV: 53.7% CMV: 24.4% HHV-6: 39% HHV-7: 39% HHV-8: 0%
Hosomi S et al., 2018 [8]	Japan	2007–2010	Retrospective	UC: 66 CD: 54 Ctrl: 29	Mucosal biopsy samples/ mucosa from surgical resected specimens	Multiplex PCR	UC HSV-1/2: 0% VZV: 0% EBV: 21.2% CMV: 15.2% HHV-6: 9.1% HHV-7: 1.5% CD HSV-1/2: 0% VZV: 0% EBV: 9.3% CMV: 0% HHV-6: 9.3% HHV-7: 3.7% Ctrl HSV-1/2: 0% VZV: 0% EBV: 0% CMV: 3.4% HHV-6: 6.9% HHV-7: 0%

CD: Crohn’s disease, CMV: cytomegalovirus, Ctrl: control, EBV: Epstein-Barr virus, HHV: human herpesvirus, HSV: herpes simplex virus, IBD: inflammatory bowel disease, LPMCs: lamina propria mononuclear cells, PCR: polymerase chain reaction, UC: ulcerative colitis, VZV: varicella-zoster virus.

## Data Availability

All the data are included in this manuscript.
